# Impact of human papillomavirus infection in semen on sperm progressive motility in infertile men: a systematic review and meta-analysis

**DOI:** 10.1186/s12958-020-00604-0

**Published:** 2020-05-07

**Authors:** Xiaodan Cao, Renxiong Wei, Xiaoxia Zhang, Jun Zhou, Jiangtao Lou, Yun Cui

**Affiliations:** Department of Clinical Laboratory, Ningbo Hospital of Traditional Chinese Medicine, Affiliated to Zhejiang Chinese Medical University, Ningbo, 315000 China

**Keywords:** Human papillomavirus, Male infertility, Sperm quality, Progressive motility, Meta-analysis

## Abstract

**Background:**

Human papillomavirus (HPV) has been considered as one of the most common sexually transmitted viruses that may be linked to unexplained infertility in men. The possible mechanisms underlying correlation between HPV infection and infertility could be related to the altered sperm parameters. Current studies have investigated the effect of HPV seminal infection on sperm quality in infertile men, but have shown inconsistent results.

**Methods:**

We systematically searched PubMed, Embase, Web of Science and CNKI for studies that examined the association between HPV seminal infection and sperm progressive motility. Data were pooled using a random-effects model. Outcomes were the sperm progressive motility rate. Results are expressed as standardised mean difference (SMD) with 95% confidence interval (CI). Heterogeneity was evaluated by the I-square (*I*^*2*^) statistic.

**Results:**

Ten studies were identified, including 616 infertile patients with HPV seminal infection and 2029 infertile controls without HPV seminal infection. Our meta-analysis results indicated that sperm progressive motility was significantly reduced in HPV-infected semen samples compared with non-infected groups [SMD:-0.88, 95% CI:-1.17 ~ − 0.59]. There existed statistical heterogeneity (*I*^*2*^ value: 86%) and the subgroup analysis suggested that study region might be the causes of heterogeneity.

**Conclusions:**

HPV semen infection could significantly reduce sperm progressive motility in infertile individuals. There were some limitations in the study such as the differences in age, sample sizes and the number of HPV genotypes detected. Further evidences are needed to better elucidate the relationship between HPV seminal infection and sperm quality.

## Introduction

Infertility is defined as the inability of a couple to conceive after 1 year of unprotected sexual intercourse, which affects approximately one-fifth of couples at the reproductive age [1]. Among them, male infertility contributes to roughly 50% of overall infertility cases [2]. Seminal infections are significant etiologic factors in male infertility and often associated with impaired semen quality [3, 4]. Chronic viral infection of the urogenital tract, especially human immunodeficiency virus (HIV) infection, may result in urethral inflammation and decreased fertility [5, 6]. Hepatitis B virus (HBV) and hepatitis C virus (HCV) infections in semen can also adversely alter seminal parameters [7, 8]. Human papillomavirus (HPV) is one of the most common sexually transmitted viruses in both males and females worldwide [9]. Some studies have reported that HPV can bind to the head of sperm and result in decreasing male fertility or even causing infertility [10]. A significant association between seminal HPV infection and male fertility abnormality has been reported [11, 12]. Also, recent researches suggested that HPV infection of semen represented a significant risk factor for infertility in men [13, 14].

The possible mechanisms underlying correlation between HPV seminal infection and infertility remain unclear [15] and one possibility is that HPV infection significantly lowered the key sperm parameters [14]. Sperm progressive motility has conventionally been considered as a good indicator of motility and a key functional parameter essential for fertilization. The effects of HPV infection on sperm progressive motility in infertile men have been investigated, but the results are controversial [16]. Several researches indicated that HPV infection was closely related to male infertility with decreased sperm progressive motility [17–25], while Zheng et al. revealed that there was no significant difference of sperm progressive motility rate between infected and non-infected infertile subjects [26]. In this research, we performed a systematic review and meta-analysis to investigate the possible impact of HPV infection in semen on sperm progressive motility in infertile individuals.

## Methods

### Literature search

Two independent reviewers searched the PubMed, Embase, Web of Science and CNKI from inception until September 2019. The study type was not restricted. The following search terms were used in combination for search strategies: “human papillomavirus”, “HPV”, “infertility”, “semen”, “sperm quality”, “sperm parameter” and “progressive motility”. We also conducted manual searches of relevant additional references cited in review articles.

### Eligibility criteria

Studies were included if sperm progressive motility could be directly extracted from the original article. Data should be expressed as mean ± standard deviation (SD). Studies were excluded if they were: 1) reports not focusing on infertile patients or participants with male accessory gland infection; 2) without SD value; 3) case reports or reviews.

The inclusion criteria of infertile patients were at least 1 year of unprotected sexual intercourse without contraception, and healthy female partners (their tubal, uterine, cervical abnormalities, and ovarian disorders were excluded). Exclusion criteria were presence of antisperm antibodies, azoospermia, undescended testis, chromosome abnormalities and history of orchitis, epididymitis, epididymo-orchitis, varicocele and/or sexually transmitted infections in couples [27]. Study populations were separated into two groups: infertile patients with HPV seminal infection and infertile patients without HPV seminal infection. Diagnosed with HPV seminal infection in general population and fertile men were also excluded.

### Data extraction and risk of bias

The data of all included articles were extracted independently by two investigators. Disagreements were discussed and resolved by consensus. Key variables of interest from each study included: first author, publication year, population characteristics (country of region, age, sample size), HPV genotype, sperm progressive motility in infertile patients with or without HPV semen infection.

The Cochrane Handbook for Systematic Reviews was used to assess the risk of bias in each study. The inclusion criteria, risk of bias at the study level and data extraction were evaluated (Supplemental Figure S1 and Supplemental Figure S2). The primary outcome was the rate of sperm progressive motility.

### Statistical analysis

The inputted data included sample sizes and outcome measures with mean and standard deviations. Outcome measures were converted into the SMD with 95% CI. Heterogeneity was evaluated by *I*^*2*^ statistic to quantify the percentage of total variation across studies. If *I*^*2*^ value was greater than 50%, the summary estimate was analyzed in a random effect model. Otherwise, a fixed effect model was used. Sensitivity analysis was conducted to estimate whether any single study influenced the stability of the meta-analytic results by sequentially removing individual included study. Publication bias was assessed by Egger’s test and statistical analyses were performed using RevMan 5.3 and STATA 16.0.

## Results

### Study characteristics

**Fig. 1:**
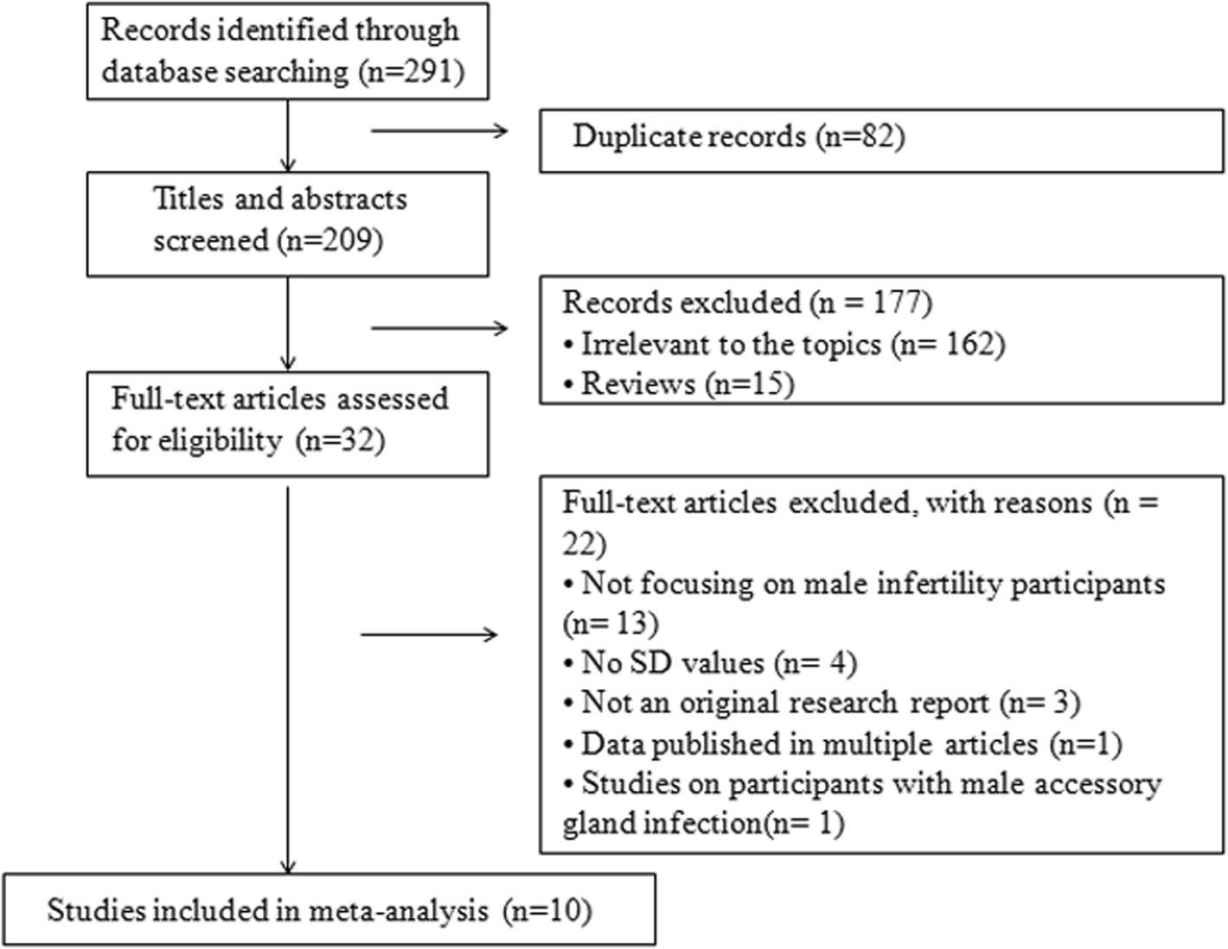
Flow diagram of the studies identified in the systematic review and meta-analysis

The initial literature search yielded 291 potentially relevant studies. Most ineligible studies were excluded based on information in the title or abstract and the remaining 32 eligible studies were reviewed in detail. The selection process was shown in Fig. 1. As a result, ten articles were included in the final meta-analysis, providing data on 616 HPV DNA positive men among 2645 participants from 3 countries. The main characteristics of the studies included in our meta-analysis were described in Table 1.

**Table 1:** Basic characteristics of the eligible studies included in the present meta-analysis

Study	Country	Age /Sample size (n)		HPV genotype	Study Design
		HPV+patients	HPV−patients		
Moghimi 2019 [20]	Iran	NA (*n* = 8)	NA (*n* = 62)	16, 18, 31, 33, 35, 39, 45, 51, 52, 56, 58, 59	Case-control
Yang 2016 [24]	China	NA (*n* = 64)	NA (*n* = 289)	18 HPV genotypes including 16,45,52,59,18,33,68,81	Cross-sectional
Garolla 2016 [21]	Italy	NA (*n* = 54)	NA (*n* = 172)	NA	Cross-sectional
Foresta 2015 [22]	Italy	37.1 ± 7.4 (*n* = 179)	38.2 ± 8.1 (*n* = 440)	6, 11, 16, 18, 26, 31, 33, 35, 39, 40,42, 43, 44, 45, 51, 52, 53, 54, 56, 58, 59, 66, 68, 69, 71, 70, 73, 74,	Cohort
Yang 2015 [25]	China	NA (*n* = 86)	NA (*n* = 41)	6,11,16,18,31,33,45,52,56,58	Cross-sectional
Zheng 2014 [26]	China	NA (*n* = 30)	NA (*n* = 300)	6,16,18,31,33,52,58	Cross-sectional
Foresta 2013 [23]	Italy	38.8 ± 9.8 (*n* = 16)	37.5 ± 5.9 (*n* = 16)	HPV-16	Cross-sectional
Yang 2013 [19]	China	NA (*n* = 107)	NA (*n* = 508)	At least 20 HPV genotypes, including 6, 11,16, 18, 31, 33,39, 40, 42, 43,45, 51, 52, 53,54, 59, 66, 68,70, 81	Cross-sectional
Garolla 2013 [17]	Italy	35.3 ± 4.7 (*n* = 61)	34.2 ± 3.8 (*n* = 104)	6, 11, 16, 18, 26, 31, 33, 35, 39, 40, 42, 43, 44, 45, 51, 52, 53, 54, 56, 58, 59, 66, 68, 69/71, 70, 73, 74, 82.	Cross-sectional
Foresta 2010 [18]	Italy	NA (*n* = 11)	NA (*n* = 97)	6,16,18,52,53,56,61,66,70,84,90	Cross-sectional

### Meta analysis

**Fig. 2:**
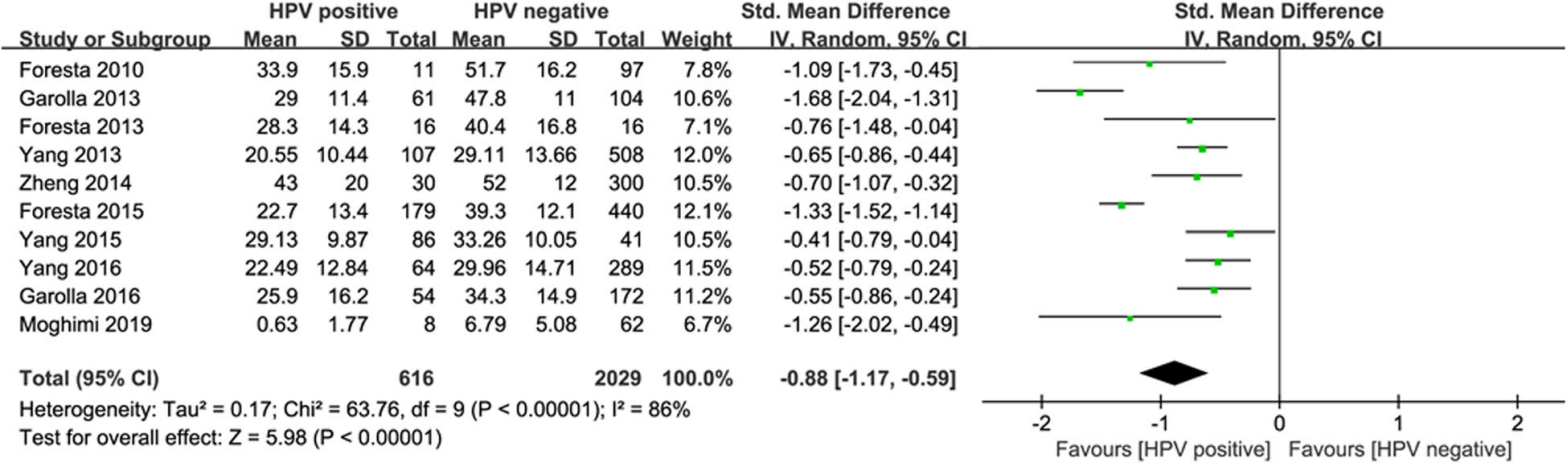
Primary outcome in overall analysis

To assess the effect of HPV seminal infection on sperm progressive motility, ten eligible studies including 616 infertile patients with HPV-infected in semen and 2029 non-infected infertile subjects were analyzed. According to the results of the heterogeneity test, the random effect model was chosen to estimate the SMD. A significant reduction of sperm progressive motility was found in semen samples of HPV-infected infertile patients compared with non-infected groups (SMD:-0.88, 95% CI:-1.17 ~ − 0.59) (Fig. 2).

**Fig. 3:**
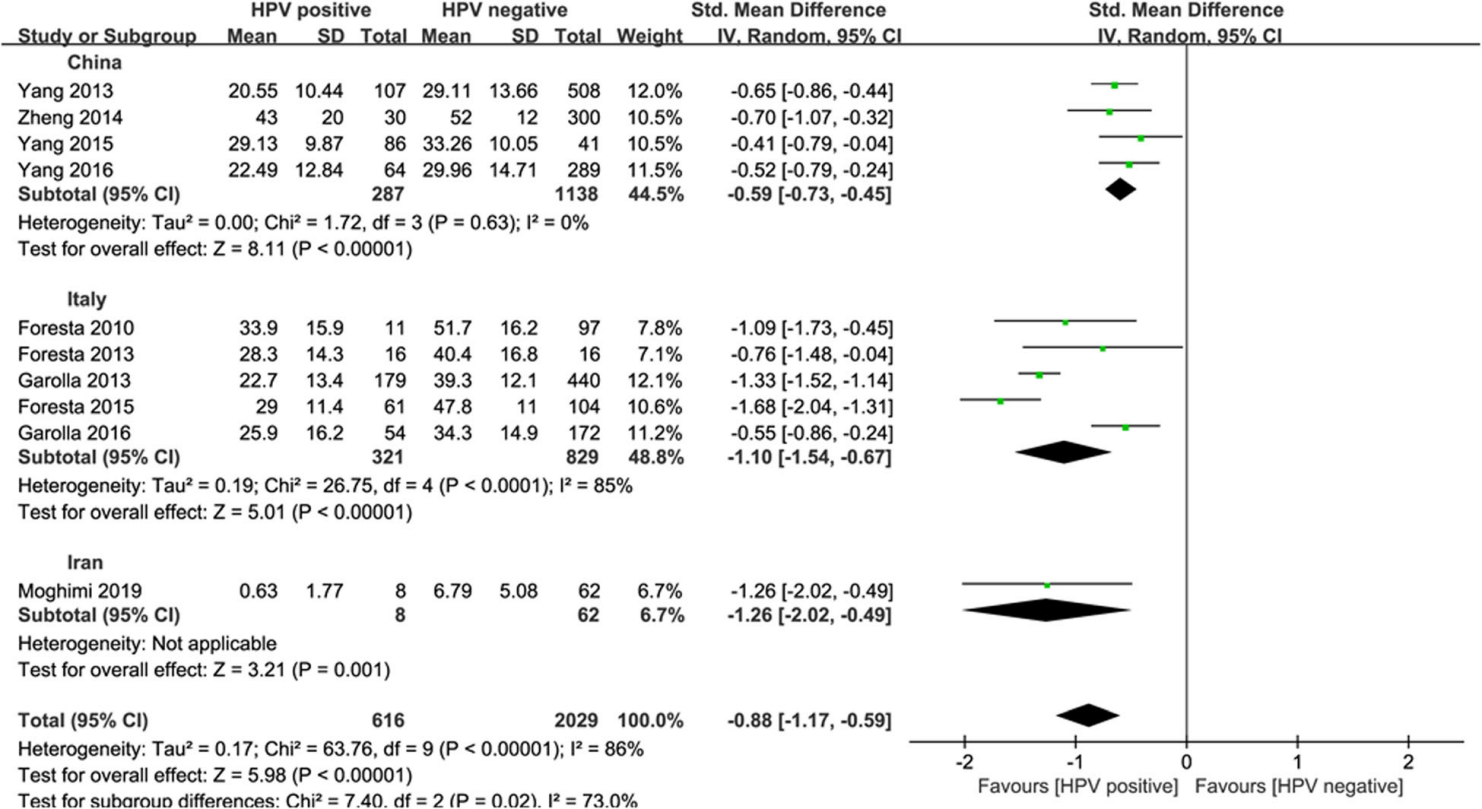
Subgroup analysis by study region

A subgroup analysis was performed to differentiate the effect size based on study region. The pooled SMD was highest in China (− 0.59, 95% CI: − 0.73 ~ − 0.45), followed by Italy (− 1.10, 95% CI: − 1.54 ~ − 0.67) and Iran (− 1.26, 95% CI: − 2.02 ~ − 0.49) (Fig. 3). There was no statistical heterogeneity in the subgroup of China.

### Sensitivity analysis

**Fig. 4:**
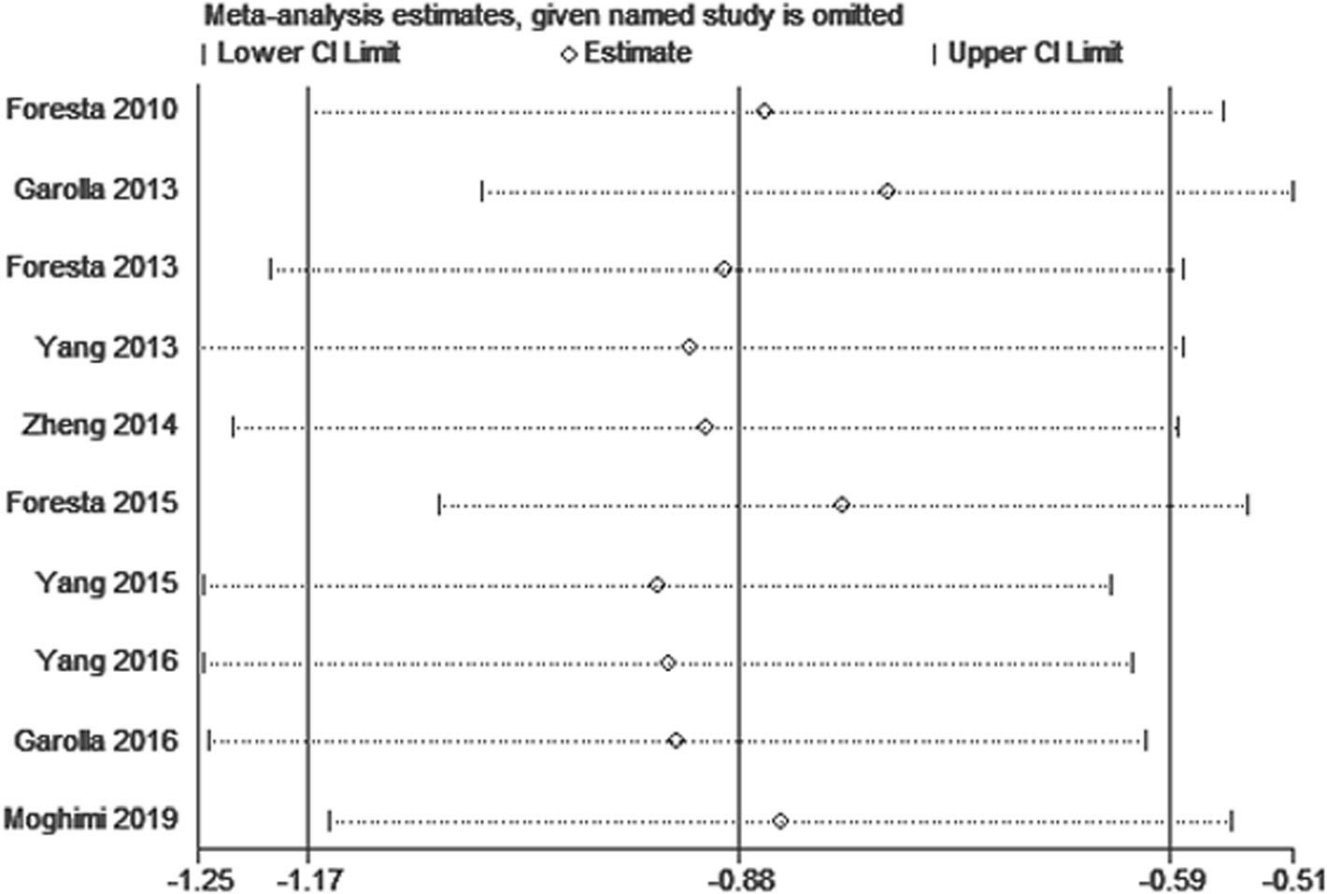
Sensitivity analysis of the association between human papillomavirus seminal infection and sperm progressive motility

None of an individual study significantly altered the overall significance of the combined SMD in the analyses relating to the impact of HPV seminal infection on sperm progressive motility in infertile individuals (Fig. 4).

### Publication bias

Egger’s test of publication bias of the seminal HPV infection on sperm progressive motility in infertile patients indicated a lack of publication bias (*P* = 0.84).

## Discussion

HPV has been considered as an infectious factor that might be linked to unexplained infertility in men. Previous meta analyses have reported the prevalence of HPV in semen [28] and the risk for male infertility [13, 14]. Laprise et al. [28] exhibited that the pooled HPV prevalence in semen was estimated at 16% for men seeking fertility evaluation/treatment and at 10% in general populations. Xiong et al. [14] and Lyu et al. [13] demonstrated that HPV semen infection was a risk factor for male fertility abnormality with an OR of 3.02 (95% CI: 2.11–4.32) and 2.93 (95% CI: 2.03–4.24) respectively. The issue whether HPV seminal infection has significance and consequence for sperm progressive motility in infertile men is controversial. The current study conducted a meta-analysis to evaluate the impact of HPV semen infection on sperm progressive motility in infertile subjects. The results showed that the prevalence of HPV detection in semen in infertile men ranged from 9.1% by Zheng et al. [26] to 67.7% by Yang et al. [25]. Sperm progressive motility reduced significantly in seminal HPV infected patients compared with non-infected groups. In the aspect of HPV genotypes distribution, the results showed that HPV-16, HPV-18/52, HPV-33, in decreasing order, were the most prevalent genotypes in semen of infertile group. Previous studies have shown that in semen the HPV were detected both in exfoliated cells [29] and in sperm surface, especially in the sperm head [17]. In an in vitro study, Carlo et al. [30] reported that HPV could infect human sperm and it localized at the equatorial region of sperm head through interaction between the HPV capsid protein L1 and syndecan-1. Moreover, HPV binding to sperm was tenacious [10, 31] and conventional methods of sperm washing could not clear HPV DNA from sperm surface [32].

The pathogenic mechanism explicating the reduction of sperm progressive motility related to seminal HPV infection might be associated with anti-sperm antibodies (ASAs), glandular dysfunction and sperm DNA fragmentation. Firstly, several studies have shown that infertile patients with HPV semen infection had a high percentage of ASAs on sperm surface and the presence of HPV in semen was frequently related with ASAs of IgA and IgG classes, which suggested that the presence of HPV DNA on the sperm surface might represent an antigenic stimulus for ASA formation [17, 33]. Although the role of ASAs is controversial, some mechanisms have been proposed affecting sperm quality: sperm agglutination and complement mediated sperm cytotoxicity occurring within the male genital tract [34]. Secondly, HPV seminal infection in infertility men may have altered proportions of secretory products mainly from prostate and seminal vesicles, which could have a negative impact on sperm motility [35]. Thirdly, HPV infection might result in the increased rate of sperm DNA fragmentation and apoptosis. In vitro study by Connelly et al. [36] indicated that sperm cells transfected with exogenous HPV E6/E7 DNA had higher percentages of breakages characteristic of apoptosis compared to the uninfected controls. In contrary, in vivo study by Cortes et al. [37] failed to find any association between HPV positive and sperm DNA fragmentation. Further evidence gathered through well-designed trials to confirm whether HPV-infected sperm is more susceptible to DNA damage is warranted.

In fact, HPV-infected sperm maintained their ability to fertilize the oocyte, interfered with implantation and embryo development, thus affecting the outcome and safety of assisted reproduction techniques (ARTs) [38]. Henneberg et al. [39] demonstrated embryo stage-specific disruption effects of HPV on early development. Perino et al. [40] reported the lower pregnancy rate and increased percentage of abortions in ARTs with HPV positive in semen. In a cross-sectional clinical study [21], cumulative pregnancy rates recorded in noninfected and infected couples undergoing ART were, respectively, 38.4 and 14.2%. During the follow-up of these pregnancies, a significantly higher miscarriage rate (62.5% vs. 16.7% of noninfected) was observed in HPV-infected subjects. In particular, all pregnancy losses of the infected group took place very early (three at 5th and two at 6th gestational week).

The results showed that *I*^*2*^*-*value was greater than 50%, which suggested that there was potential heterogeneity between studies. The heterogeneity might be attributed to differences in study region, sample size, the definition of male infertility and the number of HPV types detected. The inclusion criterion of the infertile group was at least 1 year or 2 years of unprotected sexual intercourse without conception. The study by Foresta et al. [23] included the infertile patients of case group only affected by HPV-16 semen infection and HPV-genotypes other than HPV-16 were all excluded. Multiple HPV-genotypes were detected in most of articles included in the present study and the genotype was not mentioned in one study [21]. The results of subgroup analysis showed that *I*^*2*^*-*value was equal to zero in the subgroup of China, which suggested that study region might be the causes of heterogeneity.

In addition, some limitations of the present meta-analysis should be considered when interpreting the results. Firstly, though we performed an extensive literature search, potential selection bias could not be completely avoided because only articles published in Chinese and English were included. Secondly, some important confounding factors, such as male age and environmental exposures were not always noted. These factors might have confounding effects on the correlation between HPV semen infection and reduced sperm progressive motility. Thirdly, most articles were not prospective study and might therefore decrease the reliability of our results.

## Conclusions

In summary, the current evidences suggest that HPV semen infection could significantly reduce sperm progressive motility in infertile individuals compared with non-infected infertile group. This information could make recommendations for reproduction diagnosis and treatment and could affect public health. However, this evidence is far from conclusive because of the small sample sizes and existing confounding factors of the currently available studies. Future studies with large sample size and rigorous design are necessary to elucidate the impact of HPV semen infection on sperm quality.

## Supplementary information

**Additional file 1: Figure S1.** Assessment of risk of bias.

**Additional file 2: Figure S2.** Assessment of risk of bias.

## Data Availability

The current study was based on results of relevant published studies.

## Abbreviations

ARTs: Assisted reproduction techniques
ASAs: Anti-sperm antibodies
CI: Confidence interval
HBV: Hepatitis B virus
HCV: Hepatitis C virus
HIV: Human immunodeficiency virus
HPV: Human papillomavirus
SD: Standard deviation
SMD: Standardised mean difference
