# Supplementary figures and images for: Impact of human papillomavirus infection in semen on sperm progressive motility in infertile men: a systematic review and meta-analysis
Source: Reprod Biol Endocrinol. 2020 May 7;18:38. doi: 10.1186/s12958-020-00604-0 (PMC7203819; doi:10.1186/s12958-020-00604-0)

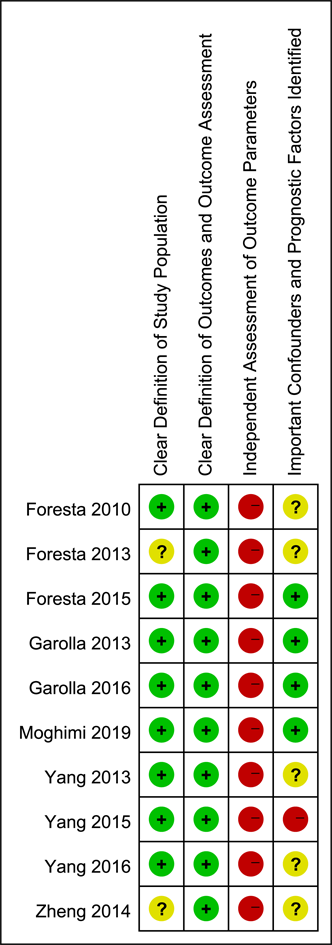

Supplement: Supplementary file 1 — Additional file 1: Figure S1. Assessment of risk of bias. [file 12958_2020_604_MOESM1_ESM.tif]

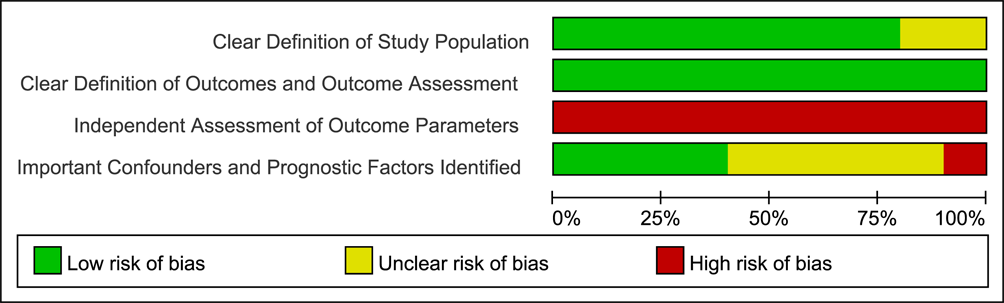

Supplement: Supplementary file 2 — Additional file 2: Figure S2. Assessment of risk of bias. [file 12958_2020_604_MOESM2_ESM.tif]
